# Elevation of Matrix Metalloproteinase-9 Level in Cerebrospinal Fluid of Tick-Borne Encephalitis Patients Is Associated with IgG Extravassation and Disease Severity

**DOI:** 10.1371/journal.pone.0077427

**Published:** 2013-11-01

**Authors:** Xiaoping Kang, Yuchang Li, Jingjing Wei, Yu Zhang, Cai Bian, Kun Wang, Xiaoyan Wu, Yi Hu, Jing Li, Yinhui Yang

**Affiliations:** 1 State Key Laboratory of Pathogen and Biosecurity, Beijing Institute of Microbiology and Epidemiology, Beijing, China; 2 Mudanjiang Forest Hospital, Mudanjiang, China; Washington University, United States of America

## Abstract

**Background:**

Tick-borne encephalitis (TBE), caused by tick-borne encephalitis virus (TBEV), is an infectious disease involving the central nervous system (CNS). The pathogenesis of CNS injury has not been clearly demonstrated. Matrix metalloproteinase-9 (MMP-9) and some cytokines, such as interleukin 6 (IL-6), may play important roles in the disruption of the blood-brain barrier (BBB) and the pathogenesis of TBE.

**Methods:**

72 cerebrospinal fluid (CSF) samples were collected from TBE patients in north eastern China. IgG levels in CSF and serum were compared and MMP-9 and IL-6 levels were evaluated by ELISA. The correlation between the elevated MMP-9 levels and IgG extravasation, disease severity, and neuroinflammation was analyzed.

**Results:**

Increased concentration of MMP-9 was detected in some of the CSF samples, and the elevation was found to be closely related to CSF TBEV IgG extravasation and enhancement of IL-6 expression. Moreover, elevated levels of MMP-9 were found to be correlated with IL-6 enhancement. Four of the 72 patients, the ones who died, presented with high CSF MMP-9 levels.

**Conclusions:**

In TBE patients, elevated CSF MMP-9 levels were associated with brain inflammatory reaction, disruption of the blood-brain barrier, and disease severity.

## Introduction

 Tick-borne encephalitis virus (TBEV), a member of Flaviviridae family, flavivirus *genus*, can infect humans through tick bites. They cause dangerous disease of the central nervous system. In China, the circulation subtype is the *far-east*. It is characterized by more pronounced neuroinvasiveness and lethality than the other subtypes, *Siberian* and *European*. Statistical data indicate that there are more than 1000 confirmed clinical cases of TBE in China each year. The main clinical symptoms of this disease are fever, headache, giddiness, and asthenia. Some cases lead to severe complications, such as meningitis, meningoencephalitis, and meningomyeloencephalitis, which are characterized by swelling of the brain due to inflammation. Though the pathogenesis of TBEV infection of the CNS is not clear, disruption of the BBB is considered to be a key event in the development of the encephalitis [1–5].

Matrix metalloproteinases (MMPs) are a family of enzymes that mediate the degradation of extracellular matrix proteins. MMPs play important roles in normal and pathological processes, including embryogenesis, wound healing, inflammation, CNS recovery and regeneration, the development of arthritis, cardiovascular diseases, pulmonary diseases, and cancer. MMP-9, a member of this family, is capable of degrading collagen IV. Multiple research groups have confirmed that MMP-9 plays a key role in the pathological progress of nerve disease [6–12]. Verma et al. evaluated the levels of MMP-9 secreted by WNV-infected endothelium and found that high levels of MMP-9 could lead to the disruption of the BBB [13]. MMP-9 can also act as an inflammatory mediator. Expression level has been found to be closely associated with the severe inflammatory reactions [14]. Applying MMP-9 inhibitor to WNV-infected mice could significantly reverse the BBB disruption and inflammatory injury in CNS [15]. For TBEV infection, though reports have confirmed that the disruption of the BBB occurs in parallel with the invasion the brain by TBEV in experimental mice, the function of MMP-9 in this process has not been clearly explored. Variations in the concentration of MMP-9 in TBE patients have not yet been fully studied [16–18]. Interleukin-6 (IL-6), an important inflammatory cytokine, is regarded as an indicator of inflammatory reaction. The correlation between MMP-9 and IL-6 levels in CNS of TBE patients has not yet been investigated.

It has been reported that leakage of the blood-brain barrier (BBB) can trigger lymphocyte infiltration and pathogen migration into the brain, which can induce severe encephalitis symptoms. IgG, which is produced in the blood, is not present in the CNS when the BBB is intact [16]. To a certain extent, IgG extravasation in the CSF may indicate disruption of the BBB. 

This paper focuses on the MMP-9 and IL-6 levels in CSF IgG-positive and IgG-negative patients. The aim of this study is to investigate the relevance between MMP-9 elevation and BBB disruption, immunopathogenesis reaction, and severity of disease in TBE patients. 

## Results

### IgG antibodies against TBEV in the CSF samples

 The concentrations of IgG antibodies against TBEV in CSF were measured using sandwich-type ELISA. Although the 72 patients presented with typical TBE symptoms and were positive for TBEV-specific IgG in serum, not all of them were positive for TBEV IgG antibodies in CSF samples. Among the 72 clinical confirmed TBE patients, only 43 had IgG-positive CSF samples. None of the control samples were found to be TBEV IgG-positive.

### Relationship between the MMP-9 Elevation and the IgG Extravasation

**Figure 1:**
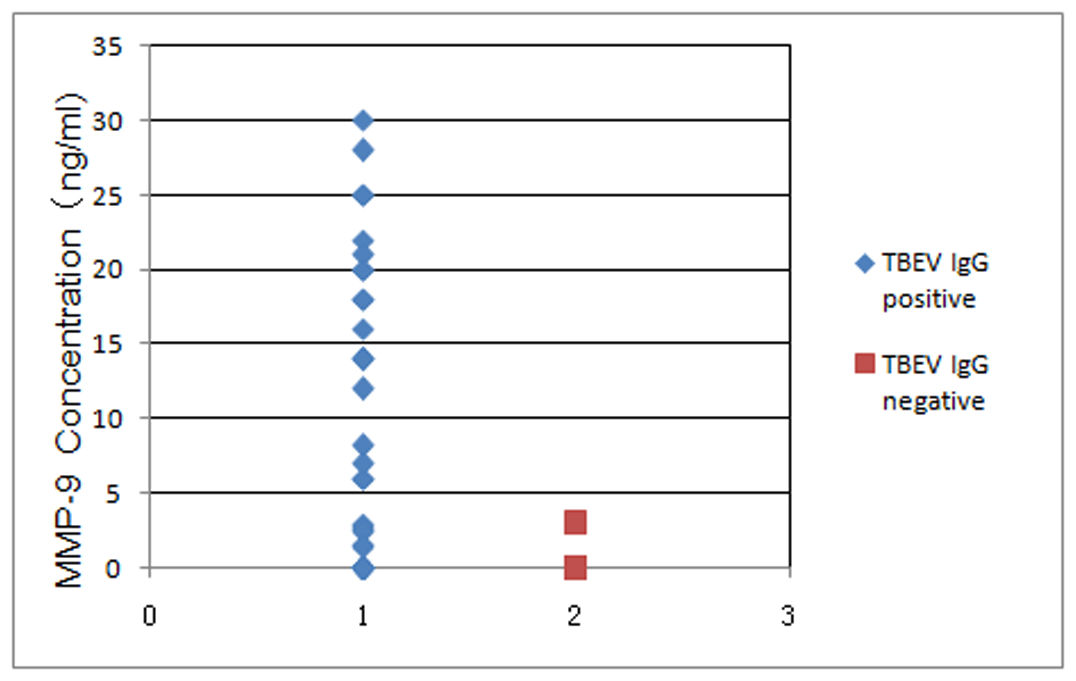
MMP-9 concentrations in TBEV IgG-positive and -negative groups. Diluted MMP-9 standards and CSF samples were added to commercial ELISA plates for MMP-9 detection. After incubation and washing, biotin-conjugated monoclonal antibody was used to bind the MMP-9 in the samples. Horseradish-peroxidase-conjugated avidin was used to amplify the signals. Then a substrate solution capable of reacting with horseradish peroxidase, was added to the wells to produce a color reaction proportional to the amount of MMP-9 present. The MMP-9 concentration in each sample was calculated by comparing it to a MMP-9 standard curve. All of the CSF samples were divided into two groups, TBEV IgG-positive samples and TBEV IgG-negative samples. The difference in the level of MMP-9 expression was analyzed using student *t* test between the two groups. The limit of detection of this MMP-9 ELISA kit was 1.25 ng/mL.

The 72 CSF samples from TBE patients were divided into two groups, a TBEV IgG-positive group and a TBEV IgG-negative group. The concentration of MMP-9 and IL-6 of each sample were analyzed and compared. In the TBEV IgG-positive group, MMP-9 levels of 14 of the 43 samples were below the detection limit. The average MMP-9 level of the remaining 29 samples was 13 ng/ml (1.4–28 ng/ml). However, only one sample in the IgG-negative group (29 samples) showed MMP-9 levels above the detection limit, which was 3 ng/ml. The MMP-9 levels of the other TBEV IgG-negative samples (28 samples) were all below the detection limit. The results are summarized in Table 1 and Figure 1, demonstrating that MMP-9 levels were significantly higher in IgG-positive samples than in IgG-negative samples (*P*<0.05).

**Table 1:** MMP-9 levels of CSF samples obtained from tick-borne encephalitis patients.

	No.of patients	MMP-9 Positives		mean concentration of MMP-9 of the positives (ng/ml)
IgG positive	43	29	13	
IgG negative	29	1	3	

### Relationship between elevation of MMP-9 and IL-6 levels

**Figure 2:**
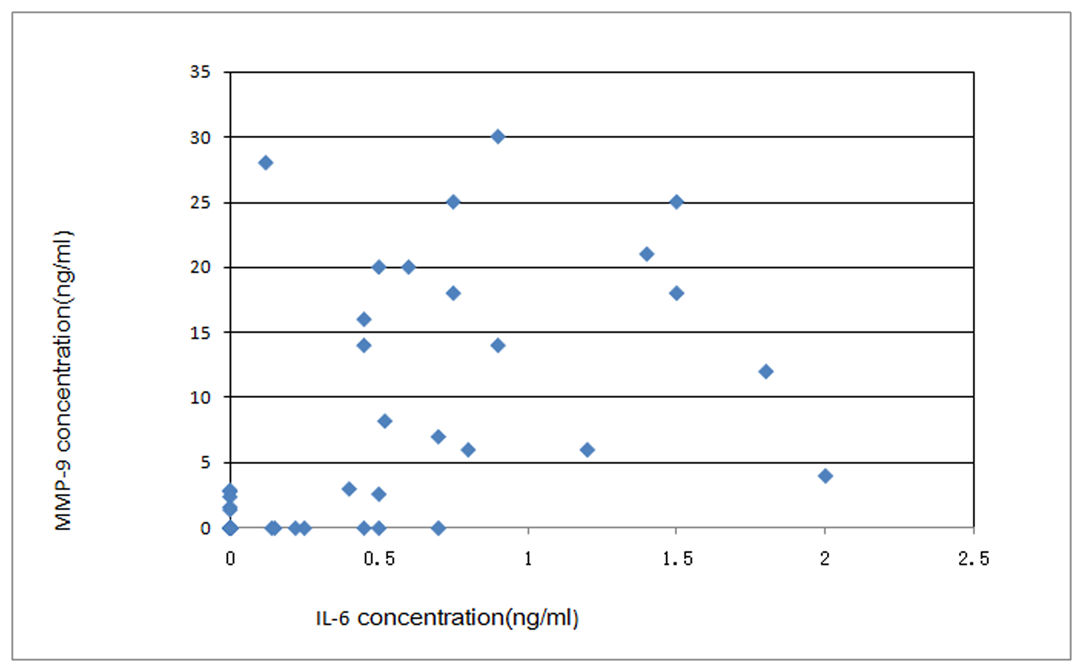
Correlations among the concentrations of MMP-9 and IL-6. The concentrations of IL-6 and MMP-9 were detected using the commercial ELISA kit, and the detection limits were 1.25 ng/mL for MMP-9 and 0.0375 ng/ml for IL-6. The correlation between the concentrations of MMP-9 and IL-6 was analyzed using Pearson statistics.

The coherence of concentration elevation between MMP-9 and IL-6 were analyzed using Pearson’ chi-square statistics. As listed in Table 2, the results demonstrated that the elevation of MMP-9 levels was consistent with that of IL-6 (*x* ^2^=30.035, *P*<0.01), which suggested that MMP-9 elevation was related to the inflammatory reaction in the brains of TBE patients. The correlation of the level of elevation of the concentrations of MMP-9 and IL-6 was analyzed using Pearson coefficient correlation calculation. The correlation between MMP-9 and IL-6 levels is presented as a scatter diagram (Figure 2), and the coefficient was calculated and found to be *r*=0.631. The results suggested that the level of elevation of IL-6 was correlated to that of MMP-9. 

**Table 2:** The correlation of elevation between MMP-9 and IL-6.

		IL-6 positive		IL-6 negative		total
MMP-9 positive		21		5		26
MMP-9 negative		7		39		46
Total		28		44		72

### Relationship between the MMP-9 elevation and the disease severity

 After hospitalization and clinical treatment, 4 patients died and the other 68 recovered. All four dead patients presented with high CSF MMP-9 levels (above 15 ng/ml), and all patients whose CSF MMP-9 levels were below the detection limit recovered well and had no complications (Table 3). 

**Table 3:** The correlation of MMP-9 elevation with disease severity.

		No.of death		No.of rerecovered		total
MMP-9 detectable		4			17	26
MMP-9 undetectable		0			46	46
	total	4			44	72

## Discussion

Although neuroinvasion of TBE virus in humans has been documented, the factors that allow an acute non-fatal febrile infection to transition to a severe and potentially fatal CNS disease are not well understood. However, the disruption of the blood-brain barrier is a key event in the pathogenesis [19,20]. 

In the present study, all of the CSF samples were collected from TBE patients early during hospitalization. Most patients presented with fever and nerve system discomfort. As the disease progressed, some patients developed severe encephalitis and four of them died. Other patients recovered completely. Seeking the factors associated with the severity of TBE is significant for clinical treatment and the prediction of progression. MMP-9 has multiple functions in CNS, including disruption of the blood-brain barrier, induction of apoptosis among neurons, participation in the development of the CNS, and recovery and regeneration [6–9]. The aim of this study was to evaluate MMP-9 levels in the CSF of TBE patients and assess any relationship between variations in MMP-9 expression and the disruption of the blood-brain barrier, inflammatory reaction in the central nervous system, and the severity of the disease.

The relationship between variations in MMP-9 expression and disruption of the blood-brain barrier disruption was evaluated by comparing the concentrations of MMP-9 in CSF TBEV IgG-positive patients to those in CSF TBEV IgG-negative patients. The results demonstrated that the phenomenon of IgG extravasation was visible in all of the MMP-9 elevated patients (only one exception), suggesting that elevated MMP-9 expression in CSF of TBE patients may be closely related to disruption of the blood-brain barrier.

Interleukin (IL)-6 is a pro-inflammatory multi-functional cytokine. In most cases, IL-6 production is associated with a protective immune response, but unregulated production can cause harm. In patients with cerebral edema attributable to brain trauma, the inflammatory reaction and some pro-inflammatory cytokines, such as IL-6 and IL-8, can be detected. An imbalance in the production of certain inflammatory cytokines, including IL-6, has been detected in the CNS of WNV patient. The role of IL-6 in both the pathogenesis and the control of virus-induced central nervous system (CNS) disease have been described on several occasions, such as WNV, HIV, and HPV, etc. [21–23]. 

As for TBEV infection, Toporkova et al showed that IL-6 levels in serum have little correlation with severity of TBE disease and TBEV IgG titration [24]. In the present study, CSF IL-6 levels were detected to analyze the relationship between MMP-9 elevation and inflammatory reaction. The results demonstrated that IL-6 elevation was accompanied by elevated MMP-9 expression, and there was a close correlation between the elevation of the levels of IL-6 and MMP-9. This suggested that, in TBE patients, the increase in MMP-9 expression was consistent with the inflammatory reaction. 

Among the 72 patients, 4 died of TBE. IgG extravasation, evaluated levels of MMP-9 and IL-6 were detected in their CSF. In contrast, none of the patients with undetectable levels of MMP-9 in the CSF died. To an extent, this indicated that MMP-9 elevation was associated with the severity of disease and may be an indicator of severe TBE. However, it was not possible to conclude that the elevated MMP-9 lead directly to death. The excess MMP-9 may contribute to the severity of the disease, but other factors, such as the type of nursing and individual capacity for recovery, may significantly influence outcome. A retrospective investigation showed that in those MMP-9 elevated patients, there were no significant difference in MMP-9 concentration among the dead, cured, and sequelae patients. 

In conclusion, it was here confirmed that increased concentrations of MMP-9 could be detected in the CSF of TBE patients. This evaluation was found to be closely associated with TBEV IgG extravasation and IL-6 elevation. All of the dead patients had elevated levels of MMP-9 expression, which suggested that, during the CNS injury caused by TBEV infection, MMP-9 elevation is closely associated with disruption of the blood-brain barrier, inflammatory reaction, and disease severity. MMP-9 might play important roles in the pathologic progress of TBEV infection. 

## Materials and Methods

### Patients Information and Ethics Statement

Seventy-two patients (*n*=72) from a TBEV circulating region in Mudanjiang, Heilongjiang Province, were hospitalized in Mudanjiang Forestry Hospital during the three May-June periods from 2010–2012, where their diagnoses of TBE were clinically confirmed. All patients had symptoms of fever and nerve discomfort and a recent history of a tick bite or bites. The concentrations of anti-TBEV IgG antibodies in the patients’ sera were all positive, as indicated by indirect fluorescence assay (IFA). No patients had a history of TBEV vaccination. All of the CSF samples were obtained during the acute phase of infection and stored at -70°C. Another 10 CSF samples from afebrile and uninfected adults with neurological disorders were taken as negative controls. These samples were collected at Beijing Chaoyang Hospital. These control adults had no recent history of visiting TBE epidemic regions or receiving TBEV vaccinations. Relevant clinical and personal details were provided by the hospitals.

This study was approved by the ethics committee of the Institute of Microbiology and Epidemiology. Each patient was informed of the purpose of the study, and a consent form was signed before sample collection.

### Detection of IgG Antibodies against TBEV in CSF

 IgG antibodies against TBEV in CSF were measured using sandwich-type ELISA. A mouse monoclonal antibody specific to TBEV was coated on the ELISA plate. Inactivated TBEV culture supernatant was added to capture the viral antigen. After incubation and washing, the CSF samples were used to bind the captured viral antigen. Horseradish-peroxidase-conjugated (HRP-conjugated) goat anti human antibody was used to detect the binding antibody in the samples. After coloration by TMB solution and measurement of the OD450 value, TBEV-specific antibodies in the CSF samples were evaluated.

### Determination of MMP-9 and IL-6 Concentrations in CSF

The MMP-9 and IL-6 concentrations in CSF from TBE patients were measured using sandwich-type ELISA kits (Boster Biological Inc., Wuhan, China). A monoclonal coating antibody was adsorbed onto polystyrene microwells to bind the target molecules in the samples or in the standards. A biotin-conjugated monoclonal antibody was used to bind the target molecules captured by the first antibody. Horseradish-peroxidase-conjugated avidin was used to amplify the signals. Then a substrate solution capable of reacting with horseradish peroxidase, was added to the wells to produce a color reaction proportional to the amount of MMP-9 and IL-6. The detection limits were 1.25 ng/mL for MMP-9 and 0.0375 ng/ml for IL-6. The MMP-9 assay recognizes both the pro- and active forms of MMP-9.

### Statistic Analysis

 After detection of TBEV IgG in CSF, all of the samples were divided into two groups, a TBEV IgG-positive group and TBEV IgG-negative group. The differences in the concentrations of MMP-9 and IL-6 were analyzed by *t* test between the two groups. *P* values less than 0.05 were considered significant. Correlations among the elevation of concentrations of MMP-9 and IL-6 were analyzed using Pearson’ chi-square test and Pearson coefficient correlation calculation. Coefficient correlation values higher than 0.5 were considered indicative of correlations. All of these statistical analyses were performed using SPSS 19 statistical software.
